# Supplementary material for: Low-affinity DNA-binding promotes cooperative activation of natural transformation in Vibrio cholerae
Source: J Bacteriol. 2026 Jun 22;208(7):e00224-26. doi: 10.1128/jb.00224-26 (PMC13316777; doi:10.1128/jb.00224-26)
Supplement: Supplemental figures and tables — Figure S1 to S5, and Tables S1 and S2. [file jb.00224-26-s0002.pdf]

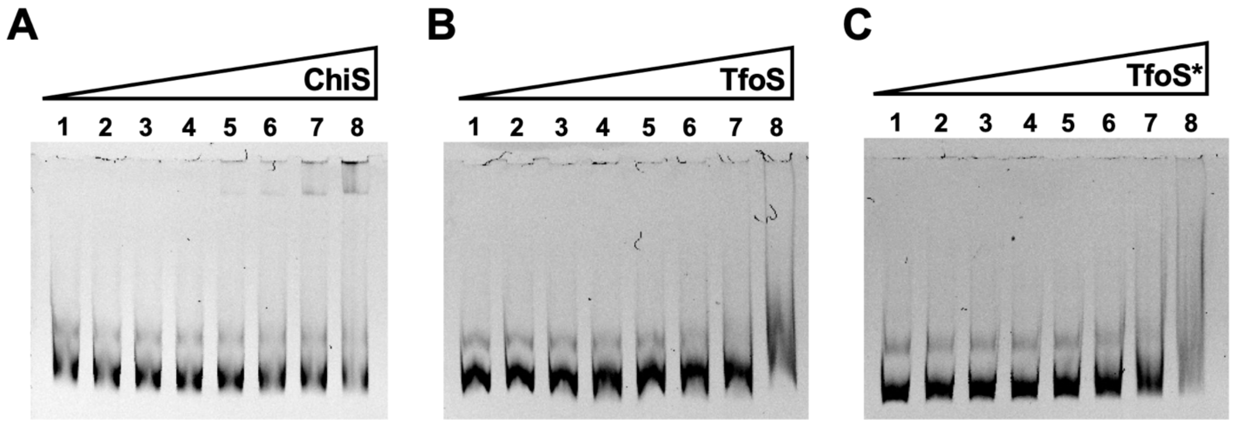

Fig. S1. *TfoS* does not alter non-specific DNA-binding activity.* EMSAs to assess the ability of the indicated purified DBD (ChiS, TfoS, or TfoS*) to bind to $P_{VCA0053}$, a promoter that is not naturally bound by any of these proteins. EMSA reactions contained increasing concentrations of the indicated protein (lane 1 = 0 nM, lane 2 = 23.4 nM, lane 3 = 46.9 nM, lane 4 = 93.8 nM, lane 5 = 187.5 nM, lane 6 = 375 nM, lane 7 = 750 nM, lane 8 = 1500 nM) and a fixed concentration (1 nM) of a Cy5-labeled $P_{VCA0053}$ DNA probe. Data are representative of three independent experiments.

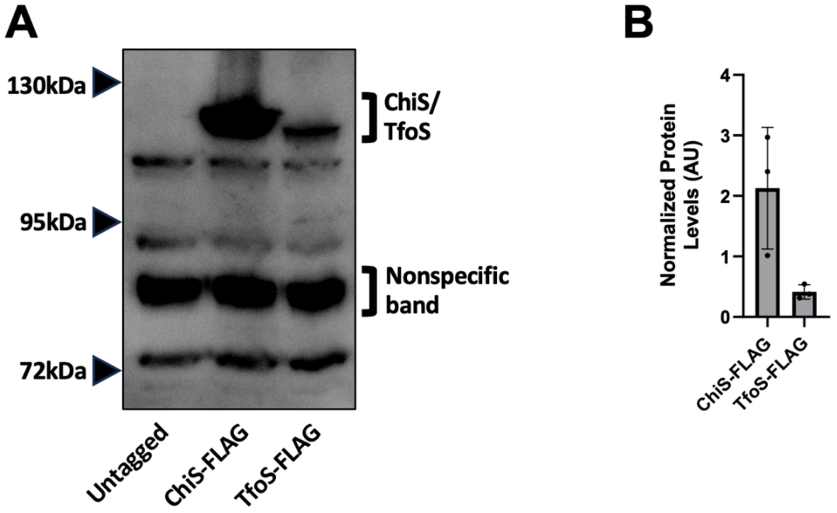

Fig. S2. *ChiS protein levels are ~5-fold greater than TfoS in vivo.* Western blots to assess the relative steady state levels of functional internally FLAG-tagged alleles of ChiS (ChiS$^{E566::FLAG}$ 129 kDa; functionality demonstrated in Klancher et al. 2020) and TfoS (TfoS$^{N929::FLAG}$ 128 kDa; see **Fig. S5** for functionality data). **(A)** A representative western blot of the indicated strains. The ChiS/TfoS band is demarcated, as well as the nonspecific band used for normalization. **(B)** Quantification of ChiS-FLAG and TfoS-FLAG levels from western blots (as in **A**). For each replicate, the normalized ChiS/TfoS level was calculated by dividing the signal intensity of the ChiS/TfoS band by the signal intensity of the nonspecific band. Data are from three independent biological replicates and shown as the mean ± SD.

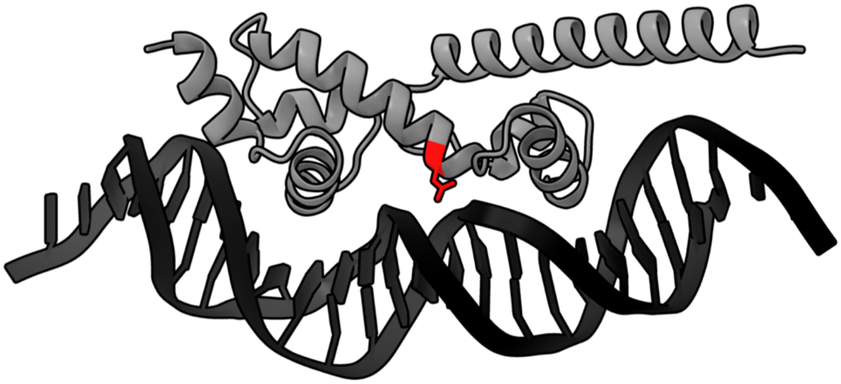

Fig. S3. The *TfoS^{E1058G} mutation is within the predicted DNA-binding domain.* An AlphaFold3 model of the TfoS DNA-binding domain in complex with DNA. The location of the E1058 residue is shown in red.

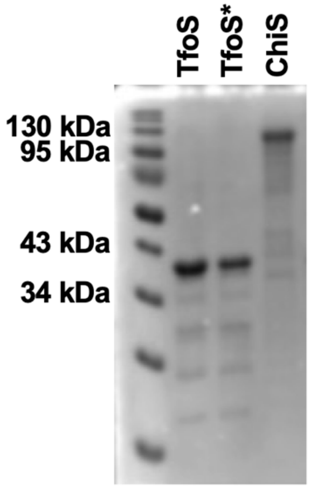

Fig. S4. *Purified ChiS and TfoS DBDs have comparable purity.* To assess purity, 37.5 picomoles of each protein was run on a 15% polyacrylamide gel and stained with Coomassie. The expected molecular weight for TfoS/TfoS* is 40.674 kDa and for ChiS is 128.831 kDa. Data are representative of two independent experiments.

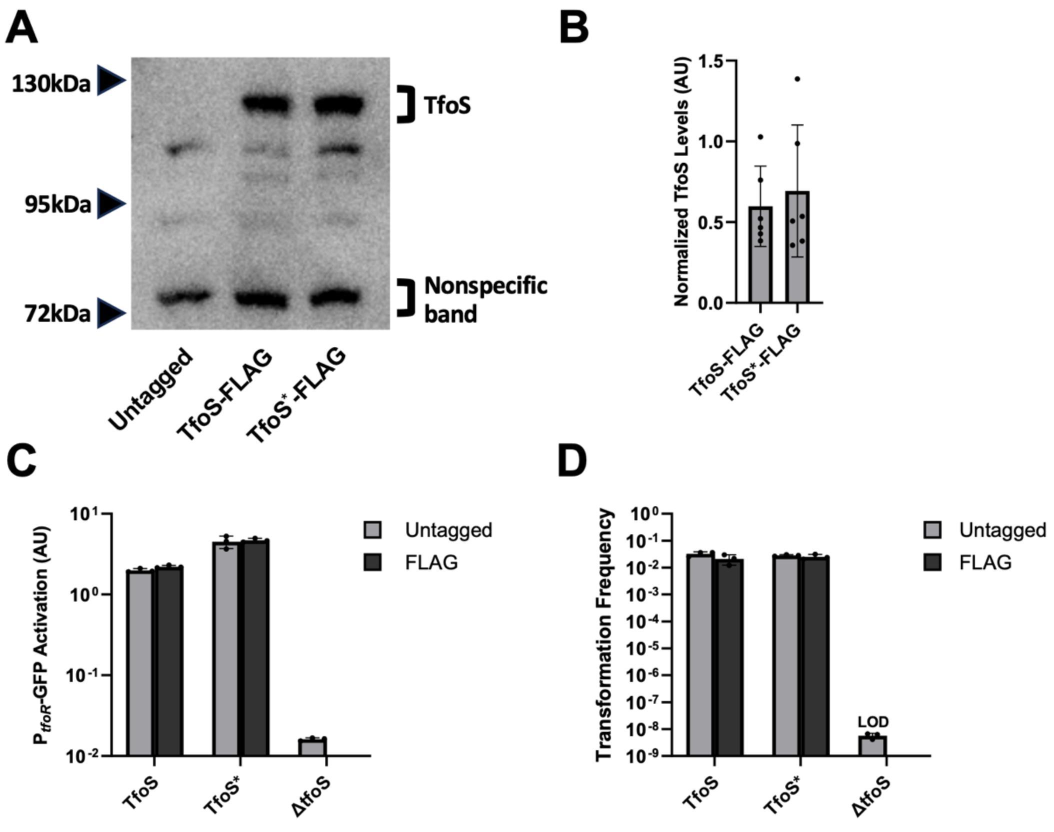

Fig. S5. *The TfoS* mutation does not affect TfoS protein levels.* Western blots and functional activity assays were performed to assess steady state levels of TfoS and to assess the functionality of the internally FLAG tagged alleles of $\text{TfoS}^{\text{N929::FLAG}}$ (internal FLAG tag inserted after position N929 in TfoS). **(A)** A representative western blot of strains containing the indicated mutations in the native copy of *tfoS*. The TfoS band is demarcated, as well as a nonspecific band that was used for normalization. **(B)** Quantification of TfoS levels from western blots (as in **A**). For each replicate, the normalized TfoS level was calculated by dividing the signal intensity of the TfoS band by the signal intensity of the nonspecific band. Data are from at least six biological replicates. **(C)** Transcriptional reporter assays to assess the functionality of the FLAG-tagged TfoS alleles. All strains harbored a P_{tfoR}-*gfp* reporter, a P_{const2}-*mTFP1* reporter, and the indicated mutations. For each replicate (*n* = 3), the geometric mean was determined by analyzing 300 individual cells. **(D)** Chitin-dependent transformation assay of the indicated strains. LOD, limit of detection. Results in **B**, **C**, and **D** are shown as the mean ± SD.

Table S1. *Strains used in this study.*

Strain ID	Genotype	Reference in Manuscript
SAD030	*V. cholerae* WT E7946 Sm^R	Parent for all *V. cholerae* strains generated in this study
ACH0529 / SAD4088	*E. coli* BL21 (DE3) harboring pGex-tev TfoS DBD $Carb^R$	Strain used for purification of TfoS DBD (residues 997-1114)
ACH0552 / SAD4089	*E. coli* BL21 (DE3) harboring pGex-tev TfoS DBD^{E1058G} $Carb^R$	Strain used for purification of TfoS DBD* (residues 997-1114)
ACH0004 / SAD3634	igVCA0265-66::$Spec^R$-P_{const2}-*mTFP1*; ΔVCA0692::Tm^R-P_{tfoR}-*gfp*; Δ*lacZ*::Kan^R-P_{chb}-*mCherry*	Fig. 2B-D<br>TfoS+ P_{tfoR} CBS+
ACH0014 / SAD3635	igVCA0265-66::$Spec^R$-P_{const2}-*mTFP1*; ΔVCA0692::Tm^R-P_{tfoR}-*gfp*; Δ*lacZ*::Kan^R-P_{chb}-*mCherry*; Δ*tfoS*::Zeo^R	Fig. 2B-D<br>ΔtfoS P_{tfoR} CBS+
ACH0748 / SAD4090	igVCA0265-66::$Spec^R$-P_{const2}-*mTFP1*; ΔVCA0692::Tm^R-$P_{tfoR}^{\Delta CBS}$-*gfp*; Δ*lacZ*::Kan^R-P_{chb}-*mCherry*; $P_{tfoR}^{\Delta CBS}$	Fig. 2B-D<br>TfoS+ ΔP_{tfoR} CBS
ACH0750 / SAD4091	igVCA0265-66::$Spec^R$-P_{const2}-*mTFP1*; ΔVCA0692::Tm^R-$P_{tfoR}^{\Delta CBS}$-*gfp*; Δ*lacZ*::Kan^R-P_{chb}-*mCherry*; $P_{tfoR}^{\Delta CBS}$; Δ*tfoS*::Zeo^R	Fig. 2B-D<br>ΔtfoS ΔP_{tfoR} CBS
ACH0423 / SAD4092	igVCA0265-66::$Spec^R$-P_{const2}-*mTFP1*; ΔVCA0692::Tm^R-P_{tfoR}-*gfp*; Δ*lacZ*::Kan^R-P_{chb}-*mCherry*; ΔVC1807::Erm^R; $TfoS^{E1058G}$	Fig. 2B-D ; Fig. S5C-D<br>TfoS* P_{tfoR} CBS+ ; TfoS* Untagged
ACH0620 / SAD4093	igVCA0265-66::$Spec^R$-P_{const2}-*mTFP1*; ΔVCA0692::Tm^R-$P_{tfoR}^{\Delta CBS}$-*gfp*; Δ*lacZ*::Kan^R-P_{chb}-*mCherry*; $P_{tfoR}^{\Delta CBS}$; ΔVC1807::Erm^R; $TfoS^{E1058G}$	Fig. 2B-D<br>TfoS* ΔP_{tfoR} CBS
TND5180 / SAD4167	Δ*lacZ*::$Spec^R$; igVC2075-76::Tm^R-P_{tfoR}-*gfp*	Fig. 3<br>$TfoS^{WT}$
ACH0645 / SAD4168	ΔVC1807::Erm^R; igVC2075-76::Tm^R-P_{tfoR}-*gfp; tfoS*^{E1058G}	Fig. 3<br>TfoS*
ACH0185 / SAD3649	igVCA0265-66::$Spec^R$-P_{const2}-*mTFP1*; Δ*lacZ*::Kan^R-P_{tfoR}-*gfp*; ΔVC1807::Cm^R; *tfoS* N929::FLAG	Fig. S5A-D<br>TfoS-FLAG
ACH0186 / SAD3650	igVCA0265-66::$Spec^R$-P_{const2}-*mTFP1*; Δ*lacZ*::Kan^R-P_{tfoR}-*gfp*; ΔVC1807::Cm^R	Fig. S5ACD<br>TfoS Untagged
ACH0510 / SAD4094	igVCA0265-66::$Spec^R$-P_{const2}-*mTFP1*; ΔVCA0692::Tm^R-P_{tfoR}-*gfp*; ΔVC1807::Cm^R; *tfoS*^{E1058G} N929::FLAG	Fig. S5A-D<br>TfoS*-FLAG
ACH0453 / SAD4095	igVCA0265-66::$Spec^R$-P_{const2}-*mTFP1*; ΔVCA0692::Tm^R-P_{tfoR}-*gfp*; Δ*tfoS*::Zeo^R	Fig. S5C-D<br>ΔtfoS
CAK1295	Δ*lacZ*::Kan^R-P_{tfoR}-*gfp*	Fig. S2<br>Untagged
TND2038	Δ*lacZ*::Kan^R-P_{tfoR}-*gfp*; ΔVC1807::Cm^R; *chiS* E566::FLAG	Fig. S2<br>ChiS-FLAG
TND1997	Δ*lacZ*::Kan^R-P_{tfoR}-*gfp*; ΔVC1807::Cm^R; *tfoS* N929::FLAG	Fig. S2<br>TfoS-FLAG

***Double identifiers under “Strain ID” refer to the same strain that has been stocked in two independent strain collections.*

Table S2. *Primers used in this study.*

**Primers for SOE Deletions**		
**Primer**	**Sequence**	**Description**
ABD123	ATTCCGGGGATCCGTCGAC	Antibiotic resistance cassette F
ABD124	TGTAGGCTGGAGCTGCTTC	Antibiotic resistance cassette R
ABD640	GCAATACACGCTGTGTTTCACCG	ΔtfoS F1 for Up arm
ABD641	gtcgacggatccccggaatCAATTCAAGAATTCGTGCTTTATTGG	ΔtfoS R1 for Up arm
ABD642	gaagcagctccagcctacaTAATAGAGCCCATCTCTATTTCATC	ΔtfoS F2 for Down arm
ABD643	TCGTTTTCTAAGGCTCGAATCGCC	ΔtfoS R2 for Down arm
BBC717	AAATAGATTTGGTGACTTTACCTCC	ΔVC1807 F1 for UP arm
ABD340	gtcgacggatccccggaatACGTTTCATTAGTCACCTCTATTGTTAA CTTGTTC	ΔVC1807 R1 for Up arm
ABD341	gaagcagctccagcctacaTAGTCGAAAATAAAAAAAAGAGGCTCG CCTC	ΔVC1807 F2 for Down arm
BBC2412	CAATTTTGCTTTTGGACCATCC	ΔVC1807 R2 for Down arm
ABD725	GAAGCAGCTCCAGCCTACA	Detect F for all deletions
CKP682	AATAGTCAACGTCAATTCTGTC	ΔtfoS detect R
BBC030	ACCAAACAATAAACGAGTAATGC	ΔVC1807 detect R
**Primers for Reporter Constructs**		
**Primer**	**Sequence**	**Description**
BBC3230	GCAGTAAATCCGACTTTGGAG	Insert construct at igVCA0265-66 F1
BBC3260	CCCGGGATCCTGTGTGAAATTG	igVCA0265-66 Spec^R R1 for P_{const2}
BBC4271	caatttcacacaggatcccgggAGGAGGTtGTGTTATGGTGAGCAAA GGTG	Amplify mTFP1 F
BBC4272	tgtaggctggagctgcttcCTATTTATACAGTTCATCCATACCATC	Amplify mTFP1 R
CKP803	gaagcagctccagcctacaTCATAATTTAAGGCGTTAGCAG	Insert construct at igVCA0265-66 F2
BBC3231	TCTCATAAACCTCATGGTAGACG	Insert construct at igVCA0265-66 R2
BBC832	GCTTTTTGCTACAACGACCG	Insert construct at VCA0692 F1
BBC263	TACCGAGGACGCGAAGCTGCTCATTAGGCACCCCAGGC	Insert construct at VCA0692 R1
ABD332	GGCTGAACGTGGTTGTCGAAAATGAC	Insert construct at *lacZ* F1
BBC219	GTTTATTTTTGTCGACTGTACAGCGTTTAAATAGAGGTCGAT ATTGACCC	Insert construct at *lacZ* R1
ABD255	gaagcagctccagcctacaCCACAATAAGCCAGAGAGCCTTAAG	Insert construct at *lacZ* F2

ABD256	CCCAAATACGGCAACTTGGCG	Insert construct at *lacZ* R2
CKP699	cagcttcgcgtcctcggtaAATAGTCAACGTCAATTCTGTC	Amplify P_{tfoR}-GFP F
BBC254	tgtaggctggagctgcttcTTAGTTGTATAGTTCATCCATGCC	Amplify P_{tfoR}-GFP R
BBC830	gaagcagctccagcctacaGTTGAGTTGGATGCAGCACC	Insert construct at VCA0692 F2
BBC834	CACAATTTCTCGCTTAAAATGTCC	Insert construct at VCA0692 R2
BBC2511	CTCATTAGGCACCCCAGGC	$P_{tfoR}^{\Delta CBS}$-GFP R1
BBC4082	gcctggggtgcctaatgagTAAAATCAACACCTTAAAAACATGATTAAGC	$P_{tfoR}^{\Delta CBS}$-GFP F2
**Primers for TfoS Constructs**		
**Primer**	**Sequence**	**Description**
ABD640	GCAATACACGCTGTGTTTCACCG	Amplify native TfoS F1
ABD643	TCGTTTTCTAAGGCTCGAATCGCC	Amplify native TfoS R2
CKP760	tccaccacttccacctgcATTTCTCACTTCAAGTTGTATGG	Insert tag after TfoS N929 R1
BBC2274	gcaggtggaagtggtggagattataaggatgacgatgacaaagcaggtggagcaggtgga	1X FLAG F
BBC2275	tccacctgctccacctgctttgtcatcgtcatccttataatctccaccacttccacctgc	1X FLAG R
CKP761	gcaggtggagcaggtggaGCGCAGGCGGTTATCGTATTTTC	Insert tag after TfoS N929 F2
AHP0018	CCTCATTTAACGAacCTTTGAAGGTTTTTC	TfoS E1058G R1
AHP0017	GAAAAACCTTCAAAGgtTCGTTAAATGAGG	TfoS E1058G F2
AHP0019	TCTAAGCGAACCTCATTTAACGgac	TfoS E1058G detect R
**Primers for TfoR Constructs**		
**Primer**	**Sequence**	**Description**
BBC4983	GTGATACCACAAGCAACTATACC	TfoR F1
BBC4980	gcttaatcatgtttttaaggtgttgattttaACCTAAGCAAACATTGTTTTCG	Delete CBS in PtfoR R1
BBC4981	cgaaaacaatgtttgcttaggtTAAAATCAACACCTTAAAAACATGATTAAGC	Delete CBS in PtfoR F2
